# *Moringa oleifera* Leaf Infusion as a Functional Beverage: Polyphenol Content, Antioxidant Capacity, and Its Potential Role in the Prevention of Metabolopathies

**DOI:** 10.3390/life15040636

**Published:** 2025-04-11

**Authors:** Gustavo A. Hernández-Fuentes, Carmen A. Sanchez-Ramirez, Salma I. Cortes-Alvarez, Alejandrina Rodriguez-Hernández, Ana O. Cabrera-Medina, Norma A. Moy-López, Jorge Guzman-Muñiz, Idalia Garza-Veloz, Iram P. Rodriguez-Sanchez, Margarita L. Martinez-Fierro, Jorge J. Álvarez-Barajas, Nadia Y. Cortes-Alvarez, Silvia G. Ceballos-Magaña, Carmen Meza-Robles, Iván Delgado-Enciso

**Affiliations:** 1 Department of Molecular Medicine, School of Medicine, University of Colima, Colima 28040, Mexico; ghfuentes@ucol.mx (G.A.H.-F.); scortes4@ucol.mx (S.I.C.-A.); arodrig@ucol.mx (A.R.-H.); aaocabrera@hotmail.com (A.O.C.-M.); 2 Colima State Institute of Cancerology, IMSS-Bienestar, Colima 28085, Mexico; carmen.qfb@gmail.com; 3 Faculty of Chemical Sciences, University of Colima, Coquimatlan 28400, Mexico; jalvarez960212@gmail.com; 4 Laboratory of Neuroscience, School of Psychology, University of Colima, Colima 28040, Mexico; moynor@ucol.mx (N.A.M.-L.); guzman72@ucol.mx (J.G.-M.); ny.cortes@ugto.mx (N.Y.C.-A.); 5 Molecular Medicine Laboratory, Unidad Académica de Medicina Humana y Ciencias de la Salud, Universidad Autónoma de Zacatecas, Zacatecas 98160, Mexico; idaliagv@uaz.edu.mx (I.G.-V.); margaritamf@uaz.edu.mx (M.L.M.-F.); 6 Molecular and Structural Physiology Laboratory, School of Biological Sciences, Universidad Autónoma de Nuevo León, San Nicolás de los Garza 66455, Mexico; iramrodriguez@gmail.com; 7 Department of Nursing and Midwifery, Division of Natural and Exact Sciences, University of Guanajuato, Guanajuato 36259, Mexico; 8 Faculty of Sciences, University of Colima, Colima 28045, Mexico; silvia_ceballos@ucol.mx; 9 Robert Stempel College of Public Health and Social Work, Florida International University, Miami, FL 33199, USA

**Keywords:** *Moringa oleifera*, infusion, polyphenols, antioxidant activity, metabolopathies, hyperlipidemia, hyperglycemia, obesity, nonalcoholic fatty liver disease

## Abstract

*Moringa oleifera* (MO) leaf infusion has gained attention for its potential therapeutic effects, particularly in metabolic health, due to its rich content of bioactive compounds, including polyphenols. The study evaluates the antioxidant properties and metabolic effects of the prophylactic administration of MO infusion in a high-fat diet (HFD)-induced murine model. First, polyphenol content (0.45 mg/g) and antioxidant activity (45.39%) were determined using Folin-Ciocalteu, DPPH, phosphomolybdenum, ferrocyanide, and anti-browning assays. In the in vivo phase, BALB/c mice were divided into three groups: a balanced diet group, a negative control group, and an HFD group supplemented with MO infusion. Over eight months, biochemical analyses, psychomotor tests, glucose tolerance assessments, and liver histopathology were conducted. MO infusion significantly reduced food intake, weight gain, lipid profiles, and liver inflammation compared to the negative control group, while promoting a metabolic profile similar to that of the balanced diet group. Additionally, it positively influenced psychomotor performance, reinforcing its neuroactive potential. These findings suggest that MO leaf infusion may serve as a functional beverage with protective effects against metabolic disorders, offering a promising natural strategy for managing obesity-related health issues.

## 1. Introduction

Metabolopathies are a group of metabolic disorders that result from dysfunctions in biochemical pathways essential for maintaining homeostasis [1]. These conditions can include obesity, diabetes, dyslipidemia, and metabolic syndrome, all of which are characterized by abnormalities in glucose and lipid metabolism [1]. According to the World Health Organization (WHO), metabolic disorders have become a major public health concern, affecting millions of people worldwide [2]. The prevalence of metabolic syndrome alone has increased significantly in recent decades, with estimates suggesting that over 25% of the global population may be affected. Individuals at higher risk include those with sedentary lifestyles, poor dietary habits, and genetic predispositions [3,4]. The clinical manifestations of these disorders vary but often include insulin resistance, hypertension, dyslipidemia, and systemic inflammation, which can lead to severe complications such as cardiovascular disease, fatty liver disease, and type 2 diabetes mellitus [3].

The etiology of metabolopathies is multifactorial, involving both genetic and environmental factors. Among the primary contributors is the excessive intake of high-fat and high-sugar diets, which promote oxidative stress and chronic inflammation [1,4]. Despite the known risk factors, metabolopathies remain a considerable health concern. Various strategies have been explored to address this issue, including alternative therapies such as traditional herbal medicine [5,6]. While many herbal remedies are documented in cultural compendiums or pharmacopoeias, most focus on treating these conditions after diagnosis. However, there are limited records of herbal remedies aimed at preventing these diseases, i.e., as a prophylactic treatment [7,8,9].

*Moringa oleifera* (MO), commonly known as moringa, is a plant recognized for its high nutritional value and bioactive compounds [10,11]. Previous studies by our research group have shown that moringa infusion provides therapeutic benefits in metabolic disorders, aiding in disease management after onset [12,13,14]. Additionally, other studies on polar extracts (methanol and ethanol) have reported positive effects on metabolic health [15,16]. Traditionally, its seeds, stems, and leaves have been used to treat various ailments [11]. Notably, moringa contains compounds from the flavonoid and phenolic acid families, such as quercetin, which exhibit strong antioxidant properties [17,18]. Phytochemical studies have also identified the presence of alkaloids, glycosides, tannins, saponins, and terpenoids, all contributing to its medicinal properties [19]. For example, niaziridin, a nitrile glycoside found in moringa extracts, has been associated with anti-inflammatory effects and attenuation of pulmonary hypertension, while moringinine, an alkaloid, has demonstrated neuroprotective potential [18,20].

One of the families that have been explored for their potential are polyphenols, especially those derived from medicinal plants, which have gained significant attention for their health benefits, particularly for conditions like cancer prevention and management [21,22]. These compounds exhibit strong antioxidant properties by neutralizing free radicals, thus reducing oxidative stress, a key factor in the development and progression of cancer [22]. They have also been shown to enhance wound healing by reducing inflammation and promoting tissue repair [22,23,24]. In metabolic syndrome, a cluster of conditions that increase the risk of heart disease, stroke, and diabetes, polyphenols help improve insulin sensitivity, reduce blood pressure, and lower cholesterol levels [25]. Their anti-inflammatory properties are also beneficial in managing chronic inflammatory diseases, such as arthritis and inflammatory bowel disease [26,27].

It is well-known that many of these compounds are incorporated into beverages to create functional foods [28]. The concept of functional beverages, which combine hydration with health-promoting properties, has gained significant attention in recent years [29]. However, the high sugar content, preservatives, and other substances in these drinks have led to some public rejection, generating a trend towards the search for more holistic, natural functional beverages [30,31]. Considering ethnomedical records, in certain regions of Mexico, people traditionally consume moringa leaf infusion as a daily beverage, suggesting potential benefits in regulating metabolic conditions, such as blood sugar control [13,32]. While moringa has been investigated for its therapeutic potential, its role in disease prevention remains underexplored. Given these potential benefits, it is essential to further investigate the role of moringa infusion in the prevention of metabolopathies. This study aims to evaluate the phytochemical profile (polyphenol content, antioxidant capacity, sugar content, chromatographic analysis) and potential protective effects of MO leaf infusion in a murine preclinical model of metabolopathies induced by a high-fat diet (HFD). It is important to note that this research is not intended to replace conventional medical treatments but rather to inform about the potential inclusion of moringa leaf infusion in future health strategies, under strict guidelines and appropriate health regulations.

## 2. Materials and Methods

### 2.1. Plant Material and Preparation of MO Infusion

MO plant material was obtained under conditions consistent with those described in our previous manuscript [33]. To ensure the reproducibility of this study and alignment with our earlier research, the same batch of MO leaves was used. The plant infusion was prepared as previously described [33], following the traditional methods used in Western Mexico. The dried MO leaves were finely ground using a hand mortar to achieve a powder texture. For the infusion, 0.7 g of the powdered leaves was mixed with 100 mL of distilled water, then heated to a boiling point (95–100 °C) and stirred for 15 min. Afterward, the infusion was filtered through cotton gauze to remove any solid particles. The filtrate was concentrated using a vacuum rotary evaporator (bath adjustment: 40 °C, rotation: 50 rpm, pressure: ∼15 psi, and condenser: 4 °C) to remove the water and subsequently freeze-dried to eliminate any water traces. The yield of the MO infusion powder was determined as a percentage (*w*/*w*) and stored in a silicone desiccator until further use. The lyophilization process was established for two key reasons: first, to obtain more consistent chemical profiles of the infusion, and second, to allow for better control over the amount of lyophilized powder for precise administration in the animal model [34,35,36]. This approach ensured more accurate dosing consistency and reliability in the infusion preparation [33]. Preliminary phytochemical characterization and chromatographic profiles, including thin-layer chromatography (TLC), infrared (IR) spectroscopy, and ^1^H nuclear magnetic resonance (NMR), were conducted under similar conditions to those reported previously [33].

### 2.2. DPPH Scavenging Activity of Polyphenols from MO Infusion

The antioxidant capacity of the MO infusion was evaluated using the DPPH radical scavenging method, a widely recognized technique offering advantages such as simplicity, rapid assessment, and reproducibility [37]. The DPPH scavenging activity of the MO infusion was assessed using a modified protocol based on the method by Lee K et al. [38], with ascorbic acid (AC) serving as the positive control [39]. Sample solutions at various concentrations (0.005–1.0 mg/mL) were mixed with DPPH solution and anhydrous ethanol. A blank sample consisting of anhydrous ethanol and DPPH solution was used for calibration. The reaction mixture was incubated in the dark for 30 min, and subsequently, the absorbance was measured at 517 nm. The scavenging activity (%) was calculated as previously described [37].

### 2.3. Polyphenol Content Using the Folin-Ciocalteu Method

The total phenolic content (TPC) of the MO infusion was measured using the Folin-Ciocalteu assay, following the protocols of Chu et al., 2022, and Chan et al., 2010 [40,41]. Triplicate samples (300 µL each) were mixed with Folin-Ciocalteu’s reagent (1.5 mL, diluted 1:10) and 1.2 mL of 7.5% (*w*/*v*) sodium carbonate, incubated for 30 min, and measured at 765 nm. This 30-min incubation time was selected based on the optimization of reaction conditions for our specific samples and was consistent with the incubation times used in other studies employing aqueous or hydroalcoholic solutions with lower sugar content [42,43]. A blank sample consisting of distilled water and Folin-Ciocalteu’s reagent, without the sample, was used to calibrate the measurements. Results were expressed as gallic acid equivalents (GAE, mg/100 g of material), using the calibration curve (y = 0.4992x − 0.4812, R^2^ = 0.9885). The MO infusion at a concentration of 1.0 mg/mL was analyzed by triplicate [41,44].

### 2.4. Anti-Browning Assay of Freshly-Cut Apple Slices

The anti-browning activity of the MO infusion was evaluated following the methodology by Lee et al., 2023, with minor adjustments [45]. Uniform apple slices (*Malus domestica* ‘Starking Delicious’) were treated with a control solvent solution (ethanol/water), a 0.5 mg/mL ascorbic acid solution (positive control) [46], or treatments with MO infusion at 1.0 mg/mL. Samples were incubated at 20 °C, and browning was recorded at 0, 12, 24, 36, and 48 h. Color changes (a*, b*, and L*) were analyzed using CorelDRAW (V. 25.0) software and the CIE color system. The overall color difference (∆E) was calculated using the formula: (∆E)^2^ = (a − a_initial)^2^ + (b − b_initial)^2^ + (L − L_initial)^2^ [45].

### 2.5. Reducing Sugar Content

A glucose calibration curve was prepared using D-glucose standards (0–500 ppm). For each standard, 4.0 mL of 500 ppm D-glucose, 1.0 mL of DNS solution, and 7.0 mL of distilled water were mixed, shaken for 1 min, heated at 80 °C for 10 min, and cooled for 20 min. For the infusion sample, 1.0 mg/mL of lyophilized material was prepared. To ensure consistency, a 2.0 mL portion of the sample solution was mixed with 1.5 mL of DNS reagent and 6.5 mL of distilled water. The mixture was shaken for 1 min, heated at 80 °C for 10 min, and cooled for 20 min with water. Absorbance was measured at 540 nm using a UV-Vis (Thermo Scientific BioMate, Waltham, MA, USA) spectrophotometer. A blank was prepared without D-glucose. To ensure reproducibility, three independent samples were prepared, and each sample was measured three times. The reducing sugar concentration in the sample was determined using the glucose calibration curve. The results are expressed in mg/mL [47]. All reagents were purchased from Sigma-Aldrich (St. Louis, MO, USA).

### 2.6. Chromatographic Analysis

The TLC analysis was performed following the method outlined by Gwatidzo et al. [25], with minor adjustments. Five 5 × 5 cm TLC plates (Silica gel 60 F254, Supelco, Bellefonte, PA, USA) were prepared, marking a pencil line 0.5 cm from one edge. Extract samples prepared at a concentration of 5.0 mg/mL were applied (2 μL) onto the pencil lines using micropipettes. For comparison purposes, three standards were utilized: quercetin (Essential Nutrition, Monterrey, Mexico), 4-methylumbelliferone, and anthrone (Sigma Aldrich, St. Louis, MO, USA), each at a concentration of 1 mg/mL [48]. The plates were placed in a development chamber and exposed to a solvent mixture of 99% ethyl acetate with 1.0% methanol. After development, the plates were removed, and the solvent front was marked with a soft pencil. The plates were then examined under UV light at 254 and 365 nm for visualization. To detect the compounds, the plates were sprayed with 1% ferric chloride (for phenol detection) and 10% aluminum chloride (AlCl_3_) for flavonoids. The plates were then photographed, and the retention factor (Rf) was calculated using the following formula: Rf = distance traveled by the spot/distance traveled by the solvent front. All TLC analyses were performed in triplicate for consistency.

The HPLC chromatographic profile of the MO infusion was analyzed using a polyphenol-based method, following protocols similar to those described by Sakakibara et al. 2003 [49] and George et al. 2015 [50]. The analysis was conducted with a Waters e2695-Alliance system equipped with a photodiode array detector (DAD) model 2998 (Waters Corporation, USA). Compound separation was performed using an XBRIDGE C18 column (150 mm × 4.6 mm, 3.5 µm particle size, Waters Corporation, Milford, MA, USA). The mobile phase was a mixture of acetonitrile and water acidified with 0.05% formic acid, and the gradient was optimized for separating target analytes. The gradient was as follows: 20% acetonitrile (0–10 min), increasing to 80% (10–40 min), maintained until 50 min, and returned to initial conditions by 60 min. The flow rate was set at 0.8 mL/min, the injection volume was 20 µL, and the column temperature was kept at 35 ± 5 °C. UV-VIS detection occurred from 200 to 400 nm, with a total run time of 60 min [48].

Reference standards, including gallic acid (GA), cinnamic acid (AC), anthrone (ANT), quercetin (Q), and 4-methylumbelliferone (4-ML), were obtained from Sigma-Aldrich (USA). HPLC-grade acetonitrile and Milli-Q water were also purchased from Sigma-Aldrich (USA). Samples were filtered before injection using Spritzen-Syringe Filters (0.22 µm pore size, Spritzen, Berlin, Germany). Weighing was performed using a high-precision analytical balance (Ohaus Corporation, Parsippany, NJ, USA). The MO infusion was injected at a concentration range of 500–1000 ppm.

### 2.7. Prophylactic Animal Model Design for MO Infusion

In this study, conditions similar to those used in a previous investigation examining the effects of Moringa oleifera (MO) were followed [33]. The current preclinical trial was conducted concurrently with another study involving groups of individuals with established disease (NAFLD) to evaluate the short-term effects of MO, the findings of which have been previously reported [33]. Due to the similarity between the present investigation and the referenced study, some values corresponding to the “control or reference groups” (healthy subjects or untreated NAFLD models) may overlap between both studies.

However, the current study incorporated the following specific adjustments: Male BALB/c mice (Envigo, Indianapolis, IN, USA), aged between 4 and 6 weeks, with an initial weight ranging from 22 to 25 g, were utilized. Each group consisted of 11 mice. The mice were housed under sterile conditions in filter-topped cages to minimize exposure to pathogens, with a maximum of 5 mice per cage. The environment was controlled with a 12 h:12 h light/dark cycle, a temperature of 23 °C, and 50% humidity. They had ad libitum access to food and water. National and international guidelines for laboratory animal care were strictly followed. Prior to the commencement of the experiment, the mice underwent a 7-day acclimatization period. All procedures adhered to national and international ethical standards for preclinical research. The study was approved by the Research Ethics Committee of the Colima State Cancer Institute, Colima, Mexico (Protocol Number: CEICC-240818-ETAMORI-010).

Animal handling followed institutional guidelines, the Mexican official norm for laboratory animals (NOM-062-ZOO-1999), and the Guide for the Care and Use of Laboratory Animals published by the National Academy of Sciences (2011) [51]. Euthanasia was performed according to the American Veterinary Medical Association (AVMA) Guidelines for the Euthanasia of Animals: 2020 Edition [52].

#### 2.7.1. Diet, Grouping, and Dosing

**Figure 1:**
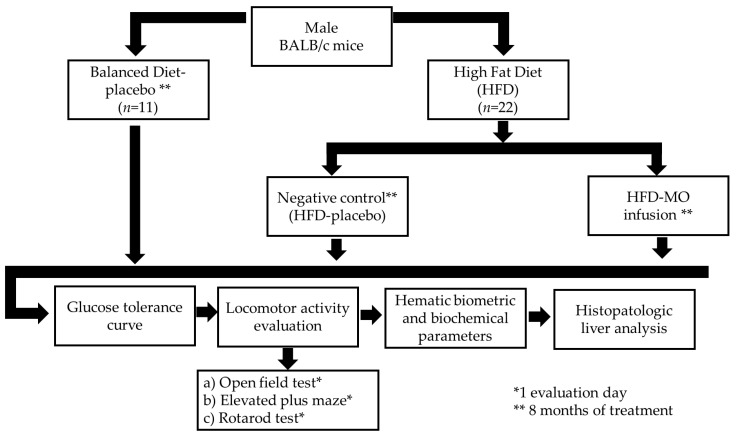
Schematic procedure for the preclinical trial.

For this study, mice were randomly assigned to three experimental groups: (1) balanced diet; (2) negative control (HFD-placebo, saline solution); and (3) HFD-MO (MO infusion). The HFD-fed model has been shown to develop NAFLD and its associated metabolic disorders within a 6-month period [53]. The balanced diet group received a balanced diet containing 6.2% net fat, 18.6% protein, 44.2% carbohydrates, 3.5% fiber, and 3.1 kcal/g (2018S Teklan and Global 18% Protein Rodent Diet, Envigo^®^, Indianapolis, IN, USA). The negative control and HFD-MO groups received a high-fat diet (HFD) with 25% net fat, 17.3% protein, 46.9% carbohydrates, 1.25% cholesterol, and 0.5% cholic acid at 4.5 kcal/g [53]. Simultaneously, with the diet, the HFD-fed mice received either placebo or MO infusion at doses of 15 mg/kg/day via stainless-steel oral gavage for 8 months (Figure 1). Twenty-four hours after the final treatment day, all animals were sacrificed by intracardiac perfusion. Blood samples were collected for biochemical analysis, and the livers were immediately removed, weighed, and processed for histopathological evaluation. The dosage of MO infusion for mice was calculated based on allometric scaling and ethnomedicinal practices. According to these practices, approximately 7 g of moringa leaves was used to prepare 1 L of water. In the current study, the equivalent dose for mice was calculated using lyophilized moringa material, with an average of 1.05 g of lyophilized powder obtained per liter. Based on this, the dose administered to the mice was adjusted to 15 mg/kg/day. This dose was provided via oral gavage at a volume of 0.2 mL per 20 g of body weight, which corresponds to the scaled equivalent of the human consumption of the infusion, while accounting for the lyophilized material used in the preparation.

#### 2.7.2. Body Weight and Food Intake

Body weight (g) was recorded on the first day of the study and then weekly for eight months using an automated electronic scale. Additionally, the daily food intake of each group was monitored weekly throughout the study period. Food intake was calculated as follows: [total food consumed per cage]/[number of mice per cage] × [number of days of food consumption] [33].

#### 2.7.3. Blood Analysis

Fasting glucose levels were measured at the start of the study (month 0) and then at months 3 and 6. A glucose tolerance test was conducted at months 6 and 8 to evaluate the progression of glycemic changes over time with the HFD and the potential protective effect of MO. Blood was collected from the tail vein for this assessment. For the intraperitoneal glucose tolerance test (IPGTT), mice were fasted overnight for 6 h prior to blood collection to ensure accurate fasting glucose measurement [33,54]. Glucose (2 mg/kg) was administered intraperitoneally, and blood glucose levels were recorded at baseline, 30, 60, 90, and 120 min post-injection. Following euthanasia, blood was obtained by cardiac puncture. EDTA-treated blood was analyzed for a complete blood count using a Beckman Coulter AC-T instrument (Beckman Coulter, Inc., Brea, CA, USA). Serum was isolated for biochemical analysis, including tests for alanine aminotransferase (SGPT), aspartate aminotransferase (SGOT), cholesterol, triglycerides (TG), total lipids, urea, and glucose using a Cobas c111 analyzer (Roche ^®^, Mexico City, Mexico).

#### 2.7.4. Histopathological Liver Analysis

Liver tissue was analyzed histopathologically as previously described [55]. A pathologist, blinded to the treatment groups, assessed liver fat accumulation and the degree of hepatic steatosis. Inflammation was evaluated based on histologic zones corresponding to oxygen supply: Zone 1 was around the portal tracts where oxygenated blood enters from the hepatic arteries; Zone 3 surrounded the central veins, where oxygen supply is limited; and Zone 2 lay between Zones 1 and 3. The inflammatory infiltrates were classified based on the extent of tissue involvement: none (0%), mild (up to 33%), moderate (33–66%), and severe (more than 66%) [56]. Histopathological analysis was conducted on digital images of the entire liver surface (both right and left lobules) at 200× magnification.

#### 2.7.5. Psychomotor Evaluation

To evaluate locomotor function, an open-field activity monitoring system was used to assess changes in activity after treatment [57]. This validated method for small rodents was performed as described previously [58]. Significant alterations in locomotor activity were considered when velocity differed substantially from the control group [59,60]. The elevated plus maze test was employed to assess the potential antianxiety effects of MO, following previously validated methods for mice [61]. The anxiety index was calculated according to Cohen et al., 2013 [59]. Balance and motor coordination were tested using the rotarod test, set at 18 rotations per minute (LE8300; Letica LSI, Pan-lab Scientific Instruments, Barcelona, Spain), recording the number of rotations and falls, as reported earlier [61].

### 2.8. Statistical Analysis

Descriptive statistics utilize mean and standard deviation. Inferential statistics involved assessing normal data distribution using the Kolmogorov-Smirnov test and confirming equality of variances through Levene’s test. Differences between groups were analyzed using either one-way ANOVA or the Kruskal-Wallis test. Post hoc analyses were conducted with either the Tukey or Mann-Whitney U tests to determine differences between each group. Statistical analyses were performed using IBM SPSS version 20 software (IBM SPSS, Chicago, IL, USA) [62], with statistical significance set at *p* < 0.05 [63].

## 3. Results

### 3.1. Antioxidant Activity and Polyphenol Content

The phytochemical profile, supported by spectroscopic and chromatographic analyses, revealed the potential composition of the MO infusion, highlighting its flavonoid content (Figures S1–S3). TLC analysis revealed distinct spots absorbing at both short (254 nm) and long wavelengths (365 nm), with Rf values around 0.3. These spots responded positively to phenol- and flavonoid-detecting reagents, confirming the presence of polyphenols (Figure S1).

IR spectroscopy detected carbonyl groups and broad O-H stretching at 3600 cm^−1^, indicative of alcohols, while functional group-specific vibrations further confirmed the presence of phenolic compounds. Aromatic rings in polyphenols showed C=C stretching vibrations between 1400–1600 cm^−1^, with more intense bands in the 1500–1600 cm^−1^ range, especially in flavonoids and multi-ring structures. The C-O stretching appeared at 1200–1300 cm^−1^ for phenolic groups and 1000–1100 cm^−1^ for ether linkages in complex polyphenols. Strong absorptions in the 600–900 cm^−1^ region corresponded to aromatic ring bending and various substituent vibrations (Figure S2). Finally, the ^1^H NMR spectroscopy revealed signals corresponding to aromatic compounds, consistent with the expected profile of polyphenols. Aromatic rings appeared between δ 6.2 and 6.8 ppm, while protons on substituted benzene rings were found between δ 6.8 and 7.5 ppm (Figure S3). The infusion appeared as a brown powder and was standardized for evaluation at a concentration of 1 mg/mL. The TPC was measured to explore the bioactive potential of the infusion. The results demonstrated that the infusion exhibited a polyphenol content of 2.56 µg/mg GAE. In terms of antioxidant capacity, the infusion displayed superior performance, inhibiting 45.39 ± 2.61% of DPPH radicals at the same concentration. Additionally, the infusion showed a concentration of reducing sugars at 0.2 mg/mL, while mild hydrolysis increased the concentration of reducing species to 0.45 mg/mL.

**Figure 2:**
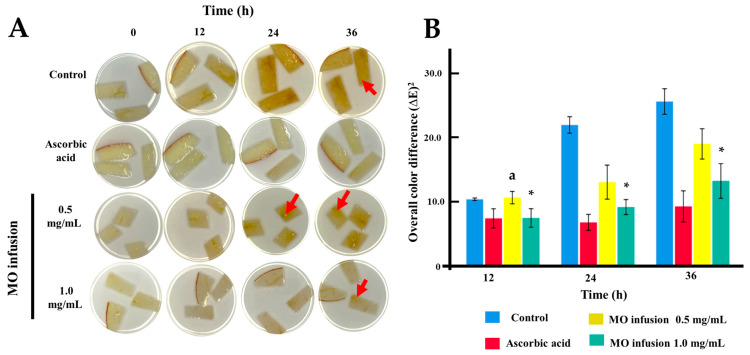
Antibrowning assay in fresh apple slices. (**A**), Images of the effect of MO infusion on the browning of freshly cut apple slices over 48 h at 20 °C. Ascorbic acid (5 mg/mL) was used as the positive control of antioxidant activity. All samples were treated in an aqueous solution. Each storage time point shows photographs of apple slices treated with MO infusion at 0.5 mg/mL and 1.0 mg/mL. The red arrows indicate softening. (**B**), Graphical representation of the overall color difference (∆E)^2^, calculated as: (∆E)^2^ = (a − a initial)^2^ + (b − b initial)^2^ + (L − L initial)^2^. * No statistical difference compared to ascorbic acid (*p* > 0.05); ^a^ No statistical difference compared to control (*p* > 0.05). Red arrows indicate browning areas.

Furthermore, the infusion was assessed for its effectiveness in an antibrowning assay on fresh apple slices stored at 20 °C for 48 h. The results, summarized in Table 1 and illustrated in Figure 2, demonstrated that the infusion at 1 mg/mL showed superior antioxidant properties, suggesting its greater potential for application. The antibrowning assay revealed varying effects of the infusion on browning at different time points (12, 24, and 36 h). At all time points, the infusion at 1.0 mg/mL showed moderate effectiveness in reducing browning. As expected, ascorbic acid consistently demonstrated the highest effectiveness in maintaining the initial color of the apple slices, with the lowest ΔE values. These findings highlight the infusion’s superior potential in preventing oxidation-related processes.

### 3.2. TLC and HPLC Analysis of the MO Infusion

The Thin Layer Chromatography (TLC) analysis revealed the presence of highly polar compounds in the infusion (Figure S1). When compared with standards such as quercetin, 4-methylumbelliferone, and anthrone, no similarities were observed in their retention factors (Rf). However, under long-wave UV light, a faint reddish-orange spot was detected with an Rf value of 0.42, showing a similarity to quercetin in retention but not in coloration pattern. Additionally, using ferric chloride as a phenolic revealer and aluminum chloride for flavonoids, a response was observed within an Rf range of 0.1 to 0.4, though with low resolution. These findings suggest the presence of phenolic compounds, but further characterization is required.

**Figure 3:**
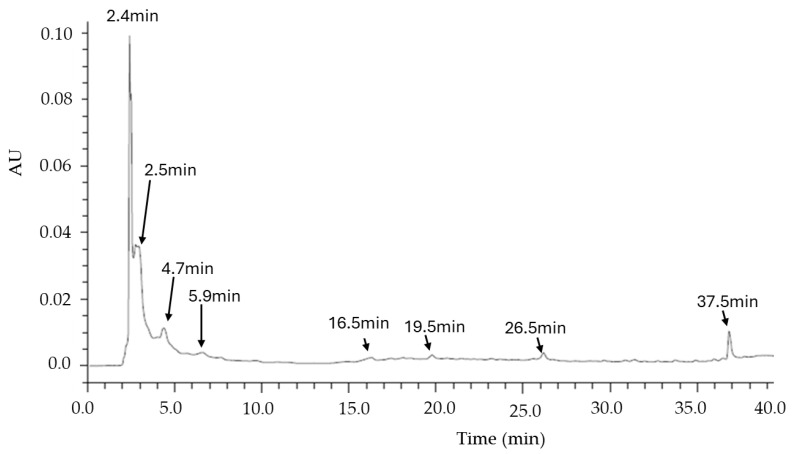
Chromatogram of MO infusion at 1000 ppm. For comparison, the chromatogram includes standards: gallic acid (GA, Rt 2.385 min), cinnamic acid (AC, Rt 30.795 min), anthrone (ANT, Rt 20.00 min), quercetin (Q, Rt 17.955 min), and 4-methylumbelliferone (4-ML, Rt 10.908 min). Chromatogram was obtained at 280 nm.

The HPLC analysis revealed distinct chemical profiles in the infusion (Figure 3). Various injection conditions were tested to optimize separation, evaluating concentrations from 50 to 1500 ppm, different gradients, temperatures between 30 and 40 °C, and pH variations. The best separation conditions were achieved at 1000 ppm, as described in the methodology. The chromatogram in Figure 3 shows intense peaks with retention times (Rt) of 2.4 to 3 min, an area under the curve (AUC) of 17,034,749, and absorbance maxima at 222 and 281 nm, as well as signals at Rt 5.9 and 7.2 min, with absorbance maxima at 222 and 291 nm and 222 and 274 nm, respectively. The injection of a set of polyphenolic metabolites (gallic acid, cinnamic acid, anthrone, quercetin, and 4-methylumbelliferone) shows that the peaks at 2.4 and 5.9 min could correspond to compounds structurally similar to gallic acid, a simple phenol commonly found in aqueous plant matrices. However, studies using higher-resolution techniques are required for confirmation. Based on previous studies, it is hypothesized that the infusion may be rich in phenolic compounds, but further analyses are required to identify these compounds and confirm their functional roles.

### 3.3. Food Intake and Weight Gain

**Figure 4:**
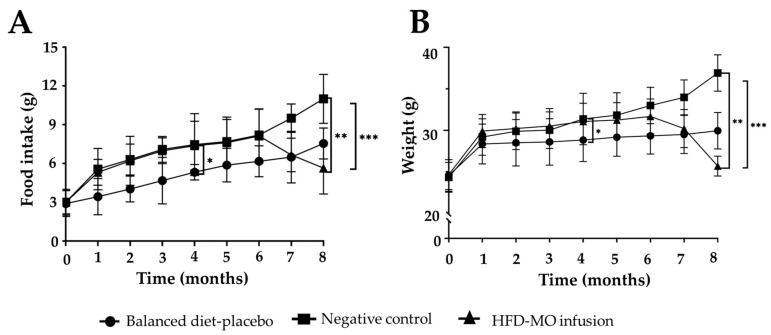
Food intake and weight gain in the groups. (**A**) Food intake. (**B**) Weight gain. All values are expressed as mean ± SEM, (*n* = 11), the differences of the Tukey’s post hoc analysis are marked when they are significant: * *p* < 0.05 when compared between negative control vs. balanced diet; ** *p* < 0.05 when compared between negative control vs. HFDMO infusion; *** *p* < 0.05 when compared between balanced diet vs. NAFLD-MO infusion.

Regarding food consumption and weight gain from the third month to the last month, it was found that the negative control group presented significantly greater weight and consumption compared to the balanced diet group (*p* < 0.001). Also, from month 2, the HFD-MO infusion group showed much lower food consumption and weight gain vs. negative control. This difference was significant until months 5 and 6, when the consumption and weight gain of the group that received the infusion was significantly lower than the group that received the HFD (*p* = 0.030 and *p* = 0.020). In the seventh month, it was shown that the group that received the infusion consumed significantly less food and had less weight gain even than the balanced diet group (*p* = 0.001). Finally, in the eighth month, the group that received MO in the form of infusion had statistically lower consumption and weight gain compared to the group with the balanced diet (*p* = 0.020). See Figure 4 and Table S1 (Supplementary Information).

### 3.4. Biochemical and Hematic Parameters

Significant reductions were observed in the biochemical parameters (total lipids, triglycerides, cholesterol, SGPT, SGOT, glucose) in the HFD-MO infusion group compared to the negative control group (*p* < 0.001). Additionally, significantly lowered levels of cholesterol, triglycerides, total lipids, and SGOT were found in the group with infusion (*p* = 0.001, in all mentioned parameters). On the other hand, regarding hematic parameters, only higher levels of hemoglobin were found in the group that received infusion compared to the balanced diet group (*p* = 0.005), as shown in Table 2.

### 3.5. Glucose Metabolism

**Figure 5:**
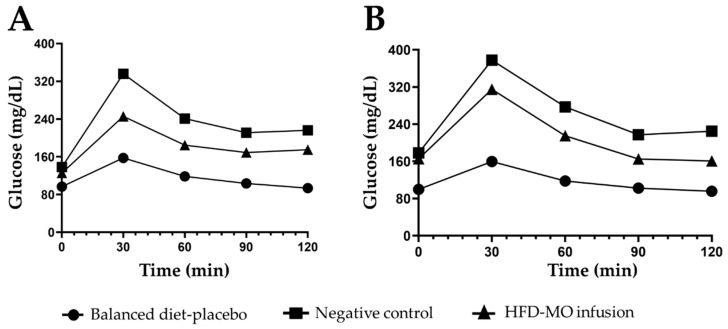
Glucose tolerance curves in the groups. (**A**) Glucose tolerance curves at 3 months. (**B**) Glucose tolerance curves at 6 months.

In the first section of the assay (zero month), basal glucose was taken, and no difference was found between the groups (*p* = 0.153). Compared with a balanced diet, the negative control group significantly increased fasting blood glucose after six months. On the glucose tolerance curve, the group with a balanced diet obtained significantly lower glucose levels compared to the negative control group (*p* ≤ 0.001) (Table 3). In months 6 and 8, the group that received MO infusion exhibited significantly better glucose tolerance than HFD mice without MO (60 min; *p* < 0.05, 90 min; *p* < 0.001, 120 min; *p* < 0.01), as can be seen in Figure 5 and Table 4.

### 3.6. Histopathological Parameters Analysis

**Figure 6:**
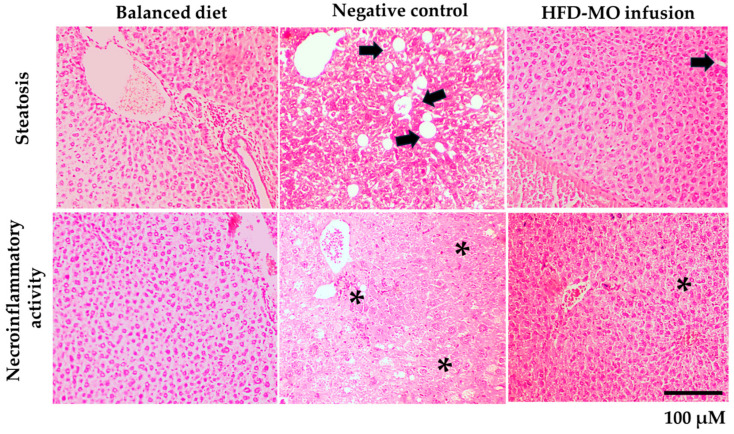
Representative images of the liver show lower steatosis and inflammation among the HFD group treated with MO infusion, compared with the HFD- placebo group. Liver tissues in the balanced diet group show no pathological changes. As an illustration, the black arrows (→) show an area of steatosis, while the asterisks (*) show the presence of inflammatory cells. Images magnified by ×200.

In terms of steatosis and inflammation (Figure 6 and Table 5), significantly lower percentages were observed in the group that received MO infusion compared to the negative control group (*p* < 0.001). Liver sections from the HFD-infused group exhibited reduced lipid accumulation and fewer inflammatory foci than those in the negative control group, suggesting a hepatoprotective effect of the infusion. Notably, the inflammatory infiltrate was markedly decreased in the MO infusion group, as evidenced by fewer lymphocytic aggregates and a more preserved hepatic architecture. The hepatocytes in this group maintained a more uniform morphology with lower cytoplasmic vacuolization and reduced ballooning degeneration compared to the negative control group.

### 3.7. Locomotor Activity Evaluation

The HFD-MO infusion group exhibited a significantly higher number of rotations compared to both the balanced diet and negative control groups (*p* = 0.003), suggesting enhanced locomotor activity. Regarding motor coordination, the HFD-MO infusion group showed a significantly lower number of falls compared to the negative control group (*p* ≤ 0.001), indicating improved neuromuscular function. Additionally, the group that received the MO infusion demonstrated a significantly lower anxiety index in comparison with both the negative control and balanced diet groups (*p* = 0.001), suggesting a potential anxiolytic effect of the infusion. These findings highlight the beneficial effects of MO infusion on motor activity and anxiety-related behavior. (Table 6).

## 4. Discussion

Unlike most studies that focus on short-term effects or therapeutic interventions, this study explores the prophylactic potential of MO leaf infusion through long-term administration. Our findings demonstrate that chronic consumption of MO infusion leads to significant benefits in weight management, metabolic regulation, and biochemical improvement, supporting its role as a preventive approach against metabolic disorders. Specifically, animals that consumed the infusion showed a notable reduction in body weight compared to the negative control group. This effect was associated with decreased food intake and overall energy consumption, suggesting a potential appetite-suppressing mechanism. Additionally, the infusion improved key biochemical markers, including total lipids, triglycerides, cholesterol, SGPT, SGOT, and glucose levels, indicating its potential for preventing non-alcoholic fatty liver disease (NAFLD), hyperlipidemia, and hyperglycemia. While previous studies have reported beneficial effects of moringa, they have largely focused on therapeutic applications rather than long-term prophylaxis [33]. Our new findings emphasize that chronic administration of MO can provide sustained metabolic benefits, enhancing glucose tolerance and contributing to better overall metabolic health. These effects are likely linked to its antioxidant properties, reinforcing the potential of moringa infusion as a natural, long-term preventive strategy for metabolic disorders.

We observed that, compared to the negative control group, the animals consuming the infusion exhibited more efficient control over weight gain, which may be related to an improvement in lipid metabolism regulation and reduced fat accumulation [64,65]. This behavior is consistent with previous studies showing that moringa has properties that promote weight regulation, such as improving the lipid profile and reducing adipogenesis [65]. In contrast, the group following a high-fat diet showed a significant increase in body weight, reflecting the direct association between an imbalanced diet and fat accumulation. These results are aligned with other research suggesting the effects of moringa in reducing obesity and improving energy metabolism [65].

The results in blood glucose levels revealed that the *Moringa oleifera* infusion has a positive effect on glucose regulation, showing a similar pattern to the balanced diet group. This finding is relevant as it suggests that the infusion could help prevent hyperglycemia and other metabolic dysfunction-related disorders [33]. While previous studies have shown that moringa can improve insulin sensitivity and reduce blood glucose levels in short-term experimental models [33], our new findings indicate that long-term consumption of MO may lead to better regulation of glucose homeostasis. This behavior, similar to that of the balanced diet group, reinforces the potential of moringa as a natural therapeutic agent for managing diabetes and other metabolic disorders [20]. Regarding the hematological parameters, the analysis of blood markers revealed no significant differences in most parameters between the groups. However, a trend towards a decrease in platelet count was observed in the group treated with the (HFD-MO) infusion compared to the other groups. Although the statistical analysis did not show a significant difference (*p* = 0.575), the potential impact on platelet counts warrants further attention. While we acknowledge the concern regarding the possible health implications, it is important to note that other hematological parameters, such as hemoglobin, hematocrit, and leukocyte count, remained stable throughout the study. Previous studies have suggested that MO may either increase platelet count [66] or exhibit anti-platelet aggregation effects [67]. This divergence in findings opens the door for future investigations to better understand the precise mechanisms through which MO influences hematological health and its broader effects on platelet function.

In histological liver analyses, the results showed that the MO infusion significantly reduced inflammation and hepatic steatosis compared to the negative control group. This finding is particularly relevant as it suggests that the infusion may have a protective effect on the liver against damage induced by high-fat diets. Furthermore, the effects observed in the infusion-treated group were similar to those of the balanced diet group, suggesting that the infusion may help mitigate liver damage associated with metabolic disturbances. Limited information regarding this topic is found in the literature, addressing the effect of MO on inflammation [16,68]. Some studies presented similar results to our study; for example, Bais et al., 2014, reported that chronic exposure to a high-fat diet (HFD) alongside supplementation with methanolic extract of MO thwarted the development of hepatic inflammation compared to non-supplemented animals [69]. On the other hand, Hamza, 2010, affirmed the hepatoprotective attributes through significant enhancement in the necroinflammatory score among animals administered with MO [68], in contrast to their counterparts. While Joung et al., 2017, demonstrated suppression of inflammation with the administration of MO in mice with an HFD [70].

In this study, we observed psychomotor effects following MO infusion consumption, which is in line with similar studies that have examined the potential neuroprotective and cognitive-enhancing properties of moringa [71]. This aligns with prior findings [13,14,33,72] suggesting that MO could be considered for managing anxiety-related disorders and potentially serve as an effective stress-relieving agent [73]. However, it would be valuable to further explore these effects using more advanced analytical or spectroscopic techniques to specifically analyze alkaloids or glucosinolates (moringin), which have also been found in the species showing protective effects against cardiovascular diseases and to participate in the removal of free radicals [15,71].

The observed effects in the laboratory models suggest that MO infusion may contain phenolic compounds, which could be responsible for its biological properties. While spectroscopic and chromatographic analyses indicate the presence of hydroxylated groups [24,49,74] and aromatic compounds [75], the specific metabolites remain unidentified. More detailed studies using techniques such as HPLC-MS or GC-MS are needed to isolate and quantify these compounds, which would allow for a more accurate attribution of the observed biological activity.

MO infusion demonstrated significant antioxidant activity in the DPPH assay and apple browning inhibition test, confirming its ability to scavenge free radicals. This antioxidant activity suggests that compounds present in the infusion may contribute to protection against oxidative stress. While the apple browning test is not directly related to the in vivo model, it serves as a complementary method to assess the antioxidative potential of MO infusion. Future studies could explore its applicability in evaluating the inhibition of other oxidative isoenzymes, such as cytochrome oxidases, monoamine oxidases, or peroxidases, which are involved in oxidative metabolism and stress responses.

There are some limitations in this study that should be addressed in future research. One key limitation is the need to explore alternative analytical methods to better understand the mechanisms behind the effects observed. While we observed promising results, particularly in liver histology and glucose metabolism, the mechanisms behind these effects are still not fully understood. Future research should delve deeper into the bioactive compounds present in moringa, especially alkaloids and other phytochemicals, by utilizing more advanced spectroscopic and analytical techniques. This would help in identifying specific compounds responsible for the observed effects and their interactions with various biological systems.

Furthermore, exploring the gut microbiota’s role in modulating the effects of MO and understanding its interaction with other pharmaceutical agents or different diets is crucial to fully comprehending its therapeutic potential. Additionally, more long-term studies are needed to assess the sustainability and broader impacts of moringa consumption in various health contexts, particularly in populations with specific health conditions such as diabetes or liver diseases. These studies should focus on clinical trials to translate these findings into human applications.

## 5. Conclusions

This study demonstrated that when administered as a prophylactic treatment, MO infusion exhibits significant biological effects, particularly in antioxidant activity, metabolic regulation, and liver health. The infusion showed strong antioxidant capacity, as evidenced by its effects on apple oxidation assays, supporting the presence of polyphenols. It did not negatively affect body weight or food intake, consistent with previous findings on moringa’s nutritional safety. Additionally, long-term consumption resulted in a metabolic profile comparable to that of a balanced diet, suggesting potential benefits in dietary interventions. Histological analysis revealed reduced liver inflammation and steatosis in the infusion-treated group, indicating hepatoprotective properties. Psychomotor performance was also positively influenced, reinforcing its reported neuroactive potential. These findings highlight the health-promoting properties of *Moringa oleifera* infusion and suggest its potential as a functional beverage. However, further studies are needed to identify the specific bioactive compounds involved and clarify their mechanisms of action.

## Figures and Tables

**Table 1:** Antibrowning results mean ± standard deviation and Post Hoc Tukey Test.

Groups		Control	Ascorbic Acid	MO Infusion 0.5 mg/mL	MO Infusion 1.0 mg/mL
Hour 12	Mean + SD	post Hoc *p*-values on hour 12			
Control	10.39 ± 0.24		<0.001	1.000	<0.001
Ascorbic acid	7.41± 1.84	<0.001		<0.001	1.000
MO infusion 0.5 mg/mL	10.65 ± 1.19	1.000	<0.001		0.004
MO infusion 1.0 mg/mL	7.47 ± 1.78	0.009	1.000	0.004	
*p* (ANOVA)	<0.001				
Hour 24	Mean + SD	post Hoc *p*-values on hour 24			
Control	21.97 ± 1.58		<0.001	<0.001	<0.001
Ascorbic acid	6.78 ± 1.53	<0.001		<0.001	0.230
MO infusion 0.5 mg/mL	13.05 ± 3.25	<0.001	<0.001		0.020
MO infusion 1.0 mg/mL	9.16 ± 1.44	<0.001	0.230	0.020	
*p* (ANOVA)	<0.001				
Hour 36	Mean + SD	post Hoc *p*-values on hour 36			
Control	25.63 ± 2.44		<0.001	<0.001	<0.001
Ascorbic acid	9.27 ± 2.98	<0.001		<0.001	0.119
MO infusion 0.5 mg/mL	19.02 ± 2.90	<0.001	<0.001		0.013
MO infusion 1.0 mg/mL	13.24 ± 3.32	<0.001	0.119	0.013	
*p* (ANOVA)	<0.001				

Mean ± standard deviation of ∆E values for the effect of MO infusion (*Moringa oleifera* infusion) on the browning of freshly cut apple slices over 36 h at 20 °C. Ascorbic acid (5 mg/mL) was used as the positive control for antioxidant activity. All samples were treated in an aqueous solution. Each storage time point includes photographs of apple slices treated with MO infusion at 0.5 and 1.0 mg/mL. Statistical significance was determined using Tukey’s post hoc test at a significance level of 0.05.

**Table 2:** Effect of MO on biochemical and hematologic parameters of the experimental groups.

Parameters	Balanced Diet	Negative Control	HFD-MO Infusion	*p* ANOVA
Cholesterol	116.9 ± 12.0	181.2 ± 15.0 ^a^	90.4 ± 16.1 ^c^	<0.001
Triglycerides	114.8 ± 15.5	185.5 ± 12.6 ^a^	87.0 ± 14.9 ^c^	<0.001
Total Lipids	156.1 ± 9.6	299.8 ± 15.1 ^a^	166.6 ± 11.3 ^c^	<0.001
SGOT	184.0 ± 14.2	403.0 ± 12.1 ^a^	159.2 ± 10.9 ^c^	<0.001
SGPT	128.9 ± 11.9	241.6 ± 16.3 ^a^	111.2 ± 12.3 ^c^	<0.001
Blood glucose	88.9 ± 13.8	141.1 ± 20.9 ^a^	94.2 ± 5.4 ^c^	0.002
Urea	11.9 ± 5.3	15.4 ± 5.8	11.4 ± 1.8	0.525
Hemoglobin	12.5 ± 1.1	14.3 ± 0.5 ^a^	14.6 ± 0.4 ^b^	<0.001
Hematocrit	35.6 ± 4.1	39.7 ± 1.5	39.7 ± 1.3	<0.001
Platelets	1207.2 ± 38.1	1210.8 ± 48.8	1085.8 ± 32.6	0.575
Leukocytes	6.4 ± 2.0	6.4 ± 2.1	6.5 ± 0.3	0.262
Erythrocytes	8.1 ± 1.0	7.7 ± 0.9	7.3 ± 0.6	0.675

All values are expressed as mean ± SEM, differences from Tukey’s post hoc analysis are marked when they are significant: ^a^ *p* ≤ 0.05 when compared balanced diet vs. Negative control group, ^b^ balanced diet vs. HFD-MO infusion, ^c^ Negative control group vs. HFD-MO infusion. (ANOVA, Tukey’s post hoc, *n* = 11 per group).

**Table 3:** Effect of MO infusion on glucose intolerance.

Glucose	Balanced Diet	Negative Control	HFD-MO Infusion	*p* ANOVA
Baseline	84.80 ± 0.8	88.60 ± 3.3	90.80 ± 3.7	0.153
3 months	94.00 ± 0.7	99.02 ± 2.5	98.50 ± 1.6	<0.001
6 months	96.70 ± 2.4	138.00 ± 2.0 ^a^	125.62 ± 2.8 ^b,c^	<0.001

All values are expressed as mean ± SEM, differences from Tukey’s post hoc analysis are marked when they are significant: ^a^ *p* ≤ 0.05 when compared balanced diet vs. Negative control group, ^b^ balanced diet vs. HFD-MO infusion, ^c^ HFD-placebo vs. HFD-MO infusion. (ANOVA, Tukey’s post hoc, *n* = 11 per group).

**Table 4:** Improved glucose tolerance by MO to the groups studied.

Minutes		Balanced Diet	Negative Control	HFD-MO Infusion	*p* ANOVA
Time 1	0	96.70 ± 2.4	138.00 ± 2.0 ^a^	125.62 ± 2.8 ^b,c^	<0.001
	30	157.60 ± 2.9	336.00 ± 2.6 ^a^	245.62 ± 3.9 ^b,c^	<0.001
	60	118.40 ± 3.2	241.00 ± 3.6 ^a^	184.62 ± 4.1 ^b,c^	<0.001
	90	103.40 ± 3.2	211.00 ± 3.6 ^a^	169.00 ± 3.9 ^b,c^	<0.001
	120	93.50 ± 3.3	216.00 ± 3.6 ^a^	175.00 ± 3.7 ^b,c^	<0.001
Time 2	0	99.57 ± 3.1	178.09 ± 3.9 ^a^	165.75 ± 2.1 ^b,c^	<0.001
	30	159.57 ± 3.1	377.81 ± 3.9 ^a^	315.12 ± 2.9 ^b,c^	<0.001
	60	117.71 ± 3.0	277.27 ± 3.9 ^a^	215.00 ± 2.9 ^b,c^	<0.001
	90	102.57 ± 3.1	217.18 ± 3.9 ^a^	165.25 ± 2.5 ^b,c^	<0.001
	120	95.57 ± 3.1	224.90 ± 3.3 ^a^	161.00 ± 2.6 ^b,c^	<0.001

All values are expressed as mean ± SEM, differences from Tukey’s post hoc analysis are marked when they are significant: ^a^ *p* ≤ 0.05 when compared balanced diet vs. Negative control group, ^b^ balanced diet vs. HFD-MO infusion, ^c^ Negative control group vs. HFD-MO infusion. ANOVA, Tukey’s post hoc, *n* = 11 per group.

**Table 5:** Liver histopathological parameters according to the groups studied.

Parameters	Balanced Diet	Negative Control	HFD-MO Infusion	*p*
Steatosis (%)	5.00 ± 3.3	68.18 ± 10.5 ^a^	8.12 ± 3.7 ^b^	<0.001 *
Necroinflammatory activity ***	0 (0–1)	3 (2–3) ^a^	1 (0–1) ^b^	<0.001 **

Values are expressed as mean ± SEM (steatosis), or median with 25th-75th percentile (Necroinflammatory activity). * ANOVA test; ** Kruskal-Wallis test. Differences from the post hoc analysis of the Tukey or Mann-Whitney U tests are marked when they are significant: ^a^ *p* ≤ 0.05 when compared balanced diet vs. HFD-placebo, ^b^ Negative control group vs. HFD-MO infusion, (*n* = 11 per group). ***: 0: none, 1: mild, 2: moderate, 3: severe.

**Table 6:** Locomotor activity evaluation of experimental groups.

Parameters	Balanced Diet	Negative Control	HFD-MO Infusion	*p* ANOVA
Number of rotations	8.85 ± 2.5	9.1 ± 3.0	17.50 ± 3.6 ^b,c^	0.003
Number of falls	17.07 ± 3.7 ^a^	21.91 ± 3.1	15.25 ± 5.0 ^c^	<0.001
Anxiety like	0.86 ± 0.09	0.86 ± 0.11	0.61 ± 0.07 ^b,c^	<0.001

All values are expressed as mean ± SEM, ^a^ *p* ≤ 0.05 when compared Balanced diet vs. Negative control group, ^b^ balanced diet vs. HFD-MO infusion, ^c^ Negative control group vs. HFD-MO infusion, (ANOVA, Tukey’s post hoc, *n* = 11 per group).

## Data Availability

The original contributions presented in the study are included in the article/Supplementary Material; further inquiries can be directed to the corresponding authors.

## Supplementary Materials

The following supporting information can be downloaded at: https://www.mdpi.com/article/10.3390/life15040636/s1, Figure S1. Thin-layer chromatography (TLC) analysis of the MO infusion (1 mg/mL), along with reference compounds. The figure displays four TLC plates visualized using different methods: (A) short-waved UV light (254 nm), (B) long-waved UV light (365 nm), (C) 10% aluminum chloride, and (D) 10% ferric chloride. The analyzed samples include the standard compounds (St): quercetin (Q, 1 mg/mL, Rf: 0.42), anthrone (Ant, 1 mg/mL, Rf: 0.95), and 4-methylumbelliferone (4-ML, 1 mg/mL, Rf: 0.85). The chromatography was performed using a solvent system of 100% ethyl acetate (AcOEt). Figure S2. FTIR or MO infusion. Figure S3. ^1^H NMR (400 MHz, DMSO-*d6*) profiles of MO infusion. Table S1. Intake and body weight of the experimental groups.

## References

[1] Lopez Entrago E., Manso Agustín V., Pazos Gonzalez A., Sanchez Celadam B. (2011). Metabolopathies. The Importance of a Good Performance Nurse. Rev. Enferm..

[2] WHO W.H.O. Global Health Observatory Data Repository. https://data.who.int/indicators/i/C6262EC/BEFA58B.

[3] Garus-Pakowska A. (2023). Metabolic Diseases—A Challenge for Public Health in the 21st Century. Int. J. Environ. Res. Public Health.

[4] Saklayen M.G. (2018). The Global Epidemic of the Metabolic Syndrome. Curr. Hypertens. Rep..

[5] Saudubray J.-M., Garcia-Cazorla À. (2018). Inborn Errors of Metabolism Overview. Pediatr. Clin. N. Am..

[6] Fahed G., Aoun L., Bou Zerdan M., Allam S., Bou Zerdan M., Bouferraa Y., Assi H.I. (2022). Metabolic Syndrome: Updates on Pathophysiology and Management in 2021. Int. J. Mol. Sci..

[7] Wachtel-Galor S., Benzie I.F.F. (2011). Herbal Medicine: An Introduction to Its History, Usage, Regulation, Current Trends, and Research Needs. Herbal Medicine: Biomolecular and Clinical Aspects.

[8] Ekor M. (2014). The Growing Use of Herbal Medicines: Issues Relating to Adverse Reactions and Challenges in Monitoring Safety. Front. Pharmacol..

[9] Xu Y., Guo W., Zhang C., Chen F., Tan H.Y., Li S., Wang N., Feng Y. (2020). Herbal Medicine in the Treatment of Non-Alcoholic Fatty Liver Diseases-Efficacy, Action Mechanism, and Clinical Application. Front. Pharmacol..

[10] Gharsallah K., Rezig L., Rajoka M.S.R., Mehwish H.M., Ali M.A., Chew S.C. (2023). *Moringa oleifera*: Processing, Phytochemical Composition, and Industrial Applications. S. Afr. J. Bot..

[11] Kou X., Li B., Olayanju J., Drake J., Chen N. (2018). Nutraceutical or Pharmacological Potential of *Moringa oleifera* Lam. Nutrients.

[12] Anwar F., Latif S., Ashraf M., Gilani A.H. (2007). *Moringa oleifera*: A Food Plant with Multiple Medicinal Uses. Phytother. Res..

[13] Olson M.E.O., Fahey J.W. (2011). *Moringa oleifera*: Un Árbol Multiusos Para Las Zonas Tropicales Seca. Rev. Mex. Biodivers..

[14] Leone A., Spada A., Battezzati A., Schiraldi A., Aristil J., Bertoli S. (2016). *Moringa oleifera* Seeds and Oil: Characteristics and Uses for Human Health. Int. J. Mol. Sci..

[15] Xie J., Peng L., Yang M., Jiang W., Mao J., Shi C., Tian Y., Sheng J. (2021). Alkaloid Extract of *Moringa oleifera* Lam. Exerts Antitumor Activity in Human Non-Small-Cell Lung Cancer via Modulation of the JAK2/STAT3 Signaling Pathway. Evid. Based Complement. Altern. Med..

[16] Das N., Sikder K., Ghosh S., Fromenty B., Dey S. (2012). *Moringa oleifera* Lam. Leaf Extract Prevents Early Liver Injury and Restores Antioxidant Status in Mice Fed with High-Fat Diet. Indian J. Exp. Biol..

[17] Vongsak B., Sithisarn P., Mangmool S., Thongpraditchote S., Wongkrajang Y., Gritsanapan W. (2013). Maximizing Total Phenolics, Total Flavonoids Contents and Antioxidant Activity of *Moringa oleifera* Leaf Extract by the Appropriate Extraction Method. Ind. Crops Prod..

[18] Kusmiyati K., Rahmawati E., Waangsir F.W.F., Selasa P. (2022). Alkaloids, Flavonoids, Tannins and Saponins Contents in *Moringa oleifera* Leaves. Indones. J. Glob. Health Res..

[19] Dhongade H.K.J., Paikra B.K., Gidwani B. (2017). Phytochemistry and Pharmacology of *Moringa oleifera* Lam. J. Pharmacopunct..

[20] Wang F., Bao Y., Shen X., Zengin G., Lyu Y., Xiao J., Weng Z. (2021). Niazirin from *Moringa oleifera* Lam. Attenuates High Glucose-Induced Oxidative Stress through PKCζ/Nox4 Pathway. Phytomedicine.

[21] Manach C., Scalbert A., Morand C., Rémésy C., Jiménez L. (2004). Polyphenols: Food Sources and Bioavailability. Am. J. Clin. Nutr..

[22] Huang G., Mei X., Hu J. (2017). The Antioxidant Activities of Natural Polysaccharides. Curr. Drug Targets.

[23] Lu Y., Yeap Foo L. (2002). Polyphenolics of Salvia—A Review. Phytochemistry.

[24] Moharram F.A., Marzouk M.S., El-Shenawy S.M., Gaara A.H., El Kady W.M. (2012). Polyphenolic Profile and Biological Activity of *Salvia splendens* Leaves. J. Pharm. Pharmacol..

[25] Gasmi A., Mujawdiya P.K., Noor S., Lysiuk R., Darmohray R., Piscopo S., Lenchyk L., Antonyak H., Dehtiarova K., Shanaida M. (2022). Polyphenols in Metabolic Diseases. Molecules.

[26] Zhang S., Xu M., Zhang W., Liu C., Chen S. (2021). Natural Polyphenols in Metabolic Syndrome: Protective Mechanisms and Clinical Applications. Int. J. Mol. Sci..

[27] Makhtoomi M., Shateri Z., Mashoufi A., Nouri M., Honarvar B., Keshani P. (2024). The Association between Dietary Polyphenol Intake and the Odds of Metabolic Syndrome. Sci. Rep..

[28] Das A.B., Goud V.V., Das C. (2019). Phenolic Compounds as Functional Ingredients in Beverages. Value-Added Ingredients and Enrichments of Beverages.

[29] Dini I. (2019). An Overview of Functional Beverages. Functional and Medicinal Beverages.

[30] Sugajski M., Buszewska-Forajta M., Buszewski B. (2023). Functional Beverages in the 21st Century. Beverages.

[31] Kowalska A., Leoniak K., Sołowiej B.G. (2024). Consumers’ Attitudes and Intentions toward Functional Beverages: A Lesson for Producers and Retailers. Decision.

[32] Olson M.E., Alvarado-Cárdenas L.O. (2016). ¿Dónde Cultivar El Árbol Milagro, *Moringa oleifera*, En México? Un Análisis de Su Distribución Potencial. Rev. Mex. Biodivers..

[33] Cortes-Alvarez S.I., Delgado-Enciso I., Rodriguez-Hernandez A., Hernandez-Fuentes G.A., Aurelien-Cabezas N.S., Moy-Lopez N.A., Cortes-Alvarez N.Y., Guzman-Muñiz J., Guzman-Esquivel J., Rodriguez-Sanchez I.P. (2024). Efficacy of Hot Tea Infusion vs. Ethanolic Extract of *Moringa oleifera* for the Simultaneous Treatment of Nonalcoholic Fatty Liver, Hyperlipidemia, and Hyperglycemia in a Murine Model Fed with a High-Fat Diet. J. Nutr. Metab..

[34] Bitwell C., Indra S.S., Luke C., Kakoma M.K. (2023). A Review of Modern and Conventional Extraction Techniques and Their Applications for Extracting Phytochemicals from Plants. Sci. Afr..

[35] Shaikh J.R., Patil M. (2020). Qualitative Tests for Preliminary Phytochemical Screening: An Overview. Int. J. Chem. Stud..

[36] Alonso-Castro A.J., Villarreal M.L., Salazar-Olivo L.A., Gomez-Sanchez M., Dominguez F., Garcia-Carranca A. (2011). Mexican Medicinal Plants Used for Cancer Treatment: Pharmacological, Phytochemical and Ethnobotanical Studies. J. Ethnopharmacol..

[37] Baliyan S., Mukherjee R., Priyadarshini A., Vibhuti A., Gupta A., Pandey R.P., Chang C.-M. (2022). Determination of Antioxidants by DPPH Radical Scavenging Activity and Quantitative Phytochemical Analysis of Ficus Religiosa. Molecules.

[38] Lee K.J., Oh Y.C., Cho W.K., Ma J.Y. (2015). Antioxidant and Anti-Inflammatory Activity Determination of One Hundred Kinds of Pure Chemical Compounds Using Offline and Online Screening HPLC Assay. Evid. Based Complement. Altern. Med..

[39] Beas-Guzmán O.F., Cabrera-Licona A., Hernández-Fuentes G.A., Ceballos-Magaña S.G., Guzmán-Esquivel J., De-León-Zaragoza L., Ramírez-Flores M., Diaz-Martinez J., Garza-Veloz I., Martínez-Fierro M.L. (2024). Ethanolic Extract of *Averrhoa carambola* Leaf Has an Anticancer Activity on Triple-Negative Breast Cancer Cells: An In Vitro Study. Pharmaceutics.

[40] Chu J., Ming Y., Cui Q., Zheng N., Yang S., Li W., Gao H., Zhang R., Cheng X. (2022). Efficient Extraction and Antioxidant Activity of Polyphenols from *Antrodia cinnamomea*. BMC Biotechnol..

[41] Chan E.W.C., Lim Y.Y., Chong K.L., Tan J.B.L., Wong S.K. (2010). Antioxidant Properties of Tropical and Temperate Herbal Teas. J. Food Compos. Anal..

[42] Schorn P.J. (1993). The European Pharmacopoeia. Med. Secoli.

[43] Hudz N., Yezerska O., Shanajda M., Horčinová Sedláčková V., Wieczorek P.P. (2019). Application of the Folin-Ciocalteu Method to the Evaluation of *Salvia sclarea* Extracts. Pharmacia.

[44] Jafri L., Saleem S., Ihsan-ul-Haq, Ullah N., Mirza B. (2017). In Vitro Assessment of Antioxidant Potential and Determination of Polyphenolic Compounds of *Hedera nepalensis* K. Koch. Arab. J. Chem..

[45] Lee J., Park H.S., Jung H.J., Park Y.J., Kang M.K., Kim H.J., Yoon D., Ullah S., Kang D., Park Y. (2023). Anti-Browning Effect of 2-Mercaptobenzo Imidazole Analogs with Antioxidant Activity on Freshly-Cut Apple Slices and Their Highly Potent Tyrosinase Inhibitory Activity. Antioxidants.

[46] Wen Y., Liang Y., Chai W., Wei Q., Yu Z., Wang L. (2021). Effect of Ascorbic Acid on Tyrosinase and Its Anti-browning Activity in Fresh-cut Fuji Apple. J. Food Biochem..

[47] Wood I.P., Elliston A., Ryden P., Bancroft I., Roberts I.N., Waldron K.W. (2012). Rapid Quantification of Reducing Sugars in Biomass Hydrolysates: Improving the Speed and Precision of the Dinitrosalicylic Acid Assay. Biomass Bioenergy.

[48] Hernandez-Fuentes G.A., Delgado-Enciso O.G., Larios-Cedeño E.G., Sánchez-Galindo J.M., Ceballos-Magaña S.G., Pineda-Urbina K., Alcalá-Pérez M.A., Magaña-Vergara N.E., Delgado-Enciso J., Díaz-Llerenas U. (2024). Comparative Analysis of Infusions and Ethanolic Extracts of *Annona muricata* Leaves from Colima, Mexico: Phytochemical Profile and Antioxidant Activity. Life.

[49] Sakakibara H., Honda Y., Nakagawa S., Ashida H., Kanazawa K. (2003). Simultaneous Determination of All Polyphenols in Vegetables, Fruits, and Teas. J. Agric. Food Chem..

[50] George V.C., Kumar D.R.N., Suresh P.K., Kumar R.A. (2015). Antioxidant, DNA Protective Efficacy and HPLC Analysis of *Annona muricata* (Soursop) Extracts. J. Food Sci. Technol..

[51] Secretaría de Agricultura y Desarrollo Rural (1999). Mexican Norm NOM 0062-ZOO-1999 Entitled Technical Specifications for the Production, Care and Use of Laboratory Animals.

[52] Leary S.R.A., Underwood W., Raymond A., Samuel C., Greenacre C., Gwaltney-Brant S., Grandin T., McCrackin M.A., Meyer R., Miller D. (2020). AVMA Guidelines for the Euthanasia of Animals: 2020 Edition.

[53] Madrigal-Perez V.M., García-Rivera A., Rodriguez-Hernandez A., Ceja-Espiritu G., Briseño-Gomez X.G., Galvan-Salazar H.R., Soriano-Hernandez A.D., Guzman-Esquivel J., Martinez-Fierro M.L., Newton-Sanchez O.A. (2015). Preclinical Analysis of Nonsteroidal Anti-Inflammatory Drug Usefulness for the Simultaneous Prevention of Steatohepatitis, Atherosclerosis and Hyperlipidemia. Int. J. Clin. Exp. Med..

[54] Fu J., Liu S., Li M., Guo F., Wu X., Hu J., Wen L., Wang J., Li X. (2024). Optimal Fasting Duration for Mice as Assessed by Metabolic Status. Sci. Rep..

[55] Garcia-Rivera A., Madriga V.M., Rodriguez-Hernandez A., Martinez-Martinez R., Martine M.L., Soriano A.D., Galvan-Salazar H.R., Gonzalez-Alvarez R., L. Valdez-Velazquez L., Espinoza-Gómez F. (2014). A Simple and Low-Cost Experimental Mouse Model for the Simultaneous Study of Steatohepatitis and Preclinical Atherosclerosis. Asian J. Anim. Vet. Adv..

[56] Haddad Y., Vallerand D., Brault A., Spénard J., Haddad P.S. (2011). NCX 1000 Alone or in Combination with Vitamin E Reverses Experimental Nonalcoholic Steatohepatitis in the Rat Similarly to UDCA. Int. J. Hepatol..

[57] Mostafavi H., Ghassemifard L., Rostami A., Alipour M., Nadri S. (2019). Trabecular Meshwork Mesenchymal Stem Cell Transplantation Improve Motor Symptoms of Parkinsonian Rat Model. Biologicals.

[58] Walf A.A., Frye C.A. (2007). The Use of the Elevated plus Maze as an Assay of Anxiety-Related Behavior in Rodents. Nat. Protoc..

[59] Cohen H., Matar M.A., Joseph Z. (2013). Animal Models of Post-Traumatic Stress Disorder. Curr. Protoc. Neurosci..

[60] Estrada-Reyes R., López-Rubalcava C., Ferreyra-Cruz O.A., Dorantes-Barrón A.M., Heinze G., Moreno Aguilar J., Martínez-Vázquez M. (2014). Central Nervous System Effects and Chemical Composition of Two Subspecies of *Agastache mexicana*; an Ethnomedicine of Mexico. J. Ethnopharmacol..

[61] Rivadeneyra-Domínguez E., Rosas-Jarquín C.J., Vázquez-Luna A., Díaz-Sobac R., Rodríguez-Landa J.F. (2019). Efecto de La Acetona Cianohidrina, Un Derivado de La Yuca, Sobre La Actividad Motora y La Función Renal y Hepática En Ratas Wistar. Neurología.

[62] Dudley W.N., Benuzillo J.G., Carrico M.S. (2004). SPSS and SAS Programming for the Testing of Mediation Models. Nurs. Res..

[63] Rosner B. (2011). Fundamentals of Biostatistics/Bernard Rosner.

[64] Almatrafi M., Vergara-Jimenez M., Murillo A., Norris G., Blesso C., Fernandez M. (2017). Moringa Leaves Prevent Hepatic Lipid Accumulation and Inflammation in Guinea Pigs by Reducing the Expression of Genes Involved in Lipid Metabolism. Int. J. Mol. Sci..

[65] Barbagallo I., Vanella L., Distefano A., Nicolosi D., Maravigna A., Lazzarino G., Di Rosa M., Tibullo D., Acquaviva R., Li Volti G. (2016). *Moringa oleifera* Lam. Improves Lipid Metabolism during Adipogenic Differentiation of Human Stem Cells. Eur. Rev. Med. Pharmacol. Sci..

[66] Seriki S. (2016). Effects of Moringa oleifera Leaves on Hematological Indices in Humans.

[67] Arabshahi-Delouee S., Aalami M., Urooj A., Krishnakantha T.P. (2009). *Moringa oleifera* Leaves as an Inhibitor of Human Platelet Aggregation. Pharm. Biol..

[68] Hamza A.A. (2010). Ameliorative Effects of *Moringa oleifera* Lam Seed Extract on Liver Fibrosis in Rats. Food Chem. Toxicol..

[69] Bais S., Singh G.S., Sharma R. (2014). Antiobesity and Hypolipidemic Activity of *Moringa oleifera* Leaves against High Fat Diet-Induced Obesity in Rats. Adv. Biol..

[70] Joung H., Kim B., Park H., Lee K., Kim H.-H., Sim H.-C., Do H.-J., Hyun C.-K., Do M.-S. (2017). Fermented *Moringa oleifera* Decreases Hepatic Adiposity and Ameliorates Glucose Intolerance in High-Fat Diet-Induced Obese Mice. J. Med. Food.

[71] Srivastava G., Ganjewala D. (2024). An Update on the Emerging Neuroprotective Potential of *Moringa oleifera* and Its Prospects in Complimentary Neurotherapy. Phytomed. Plus.

[72] Islam M.T., Martins N., Imran M., Hameed A., Ali S.W., Salehi B., Ahmad I., Hussain A., Sharifi-Rad J. (2020). Anxiolytic-like Effects of *Moringa oleifera* in Swiss Mice. Cell. Mol. Biol..

[73] Rosdy M.S., Rofiee M.S., Samsulrizal N., Salleh M.Z., Teh L.K. (2021). Understanding the Effects of *Moringa oleifera* in Chronic Unpredictable Stressed Zebrafish Using Metabolomics Analysis. J. Ethnopharmacol..

[74] Khan N., Mukhtar H. (2007). Tea Polyphenols for Health Promotion. Life Sci..

[75] Wongsa P., Phatikulrungsun P., Prathumthong S. (2022). FT-IR Characteristics, Phenolic Profiles and Inhibitory Potential against Digestive Enzymes of 25 Herbal Infusions. Sci. Rep..

