# Correction: Hernández-Fuentes et al. *Moringa oleifera* Leaf Infusion as a Functional Beverage: Polyphenol Content, Antioxidant Capacity, and Its Potential Role in the Prevention of Metabolopathies. *Life* 2025, *15*, 636

**DOI:** 10.3390/life16030447

**Published:** 2026-03-10

**Authors:** Gustavo A. Hernández-Fuentes, Carmen A. Sanchez-Ramirez, Salma I. Cortes-Alvarez, Alejandrina Rodriguez-Hernández, Ana O. Cabrera-Medina, Norma A. Moy-López, Jorge Guzman-Muñiz, Idalia Garza-Veloz, Iram P. Rodriguez-Sanchez, Margarita L. Martinez-Fierro, Jorge J. Álvarez-Barajas, Nadia Y. Cortes-Alvarez, Silvia G. Ceballos-Magaña, Carmen Meza-Robles, Iván Delgado-Enciso

**Affiliations:** 1 Department of Molecular Medicine, School of Medicine, University of Colima, Colima 28040, Mexico; ghfuentes@ucol.mx (G.A.H.-F.); scortes4@ucol.mx (S.I.C.-A.); arodrig@ucol.mx (A.R.-H.); aaocabrera@hotmail.com (A.O.C.-M.); 2 Colima State Institute of Cancerology, IMSS-Bienestar, Colima 28085, Mexico; carmen.qfb@gmail.com; 3 Faculty of Chemical Sciences, University of Colima, Coquimatlan 28400, Mexico; 4 Laboratory of Neuroscience, School of Psychology, University of Colima, Colima 28040, Mexico; moynor@ucol.mx (N.A.M.-L.); guzman72@ucol.mx (J.G.-M.); ny.cortes@ugto.mx (N.Y.C.-A.); 5 Molecular Medicine Laboratory, Unidad Académica de Medicina Humana y Ciencias de la Salud, Universidad Autónoma de Zacatecas, Zacatecas 98160, Mexico; idaliagv@uaz.edu.mx (I.G.-V.); margaritamf@uaz.edu.mx (M.L.M.-F.); 6 Molecular and Structural Physiology Laboratory, School of Biological Sciences, Universidad Autónoma de Nuevo León, San Nicolás de los Garza 66455, Mexico; iramrodriguez@gmail.com; 7 Department of Nursing and Midwifery, Division of Natural and Exact Sciences, University of Guanajuato, Guanajuato 36259, Mexico; 8 Faculty of Sciences, University of Colima, Colima 28045, Mexico; silvia_ceballos@ucol.mx; 9 Robert Stempel College of Public Health and Social Work, Florida International University, Miami, FL 33199, USA

## Error in Figure

In the original publication [1], there was a mistake in Figure 6. In the originally published version of this article, some histological micrographs in Figure 6 were inadvertently duplicated from a previous study by the same authors and labeled differently. The duplicated images corresponded to liver tissue from the Balanced diet, Negative control and HFD-MO infusion groups and were included solely as representative qualitative examples, not for quantitative evaluation.

New and correct representative images from the Balanced diet, Negative control and HFD-MO infusion groups have now been provided and inserted in place of the duplicated ones. This correction ensures that Figure 6 accurately reflects the histopathological characteristics described in the manuscript.

These changes do not affect the study’s data, analyses, results, or conclusions.

The authors sincerely apologize for this inadvertent error. The corrected Figure 6 appears below. The authors state that the scientific conclusions are unaffected. This correction was approved by the Academic Editor. The original publication has also been updated.

## Text Correction 1

There was an error in the original publication. Specifically, the omitted text that was included in Section 2.1. Plant Material and Preparation of MO Infusion was identical to that of a previous article by the same authors. The methodological description in this section originally contained text identical to that used in a previous publication by the same authors [2]. This occurred because both studies intentionally employed the same batch of Moringa oleifera (MO), the same infusion preparation, and equivalent analytical procedures to ensure reproducibility and comparability.

The section has now been rephrased to explicitly state that the infusion was prepared as previously described [1], thereby maintaining methodological accuracy while avoiding verbatim repetition.

A correction has been made to Section 2.1. Plant Material and Preparation of MO Infusion.

*2.1. Plant Material and Preparation of MO Infusion*

MO plant material was obtained under conditions consistent with those described in our previous manuscript [33]. To ensure the reproducibility of this study and alignment with our earlier research, the same batch of MO leaves was used. The plant infusion was prepared as previously described [33], following the traditional methods used in Western Mexico. The dried MO leaves were finely ground using a hand mortar to achieve a powder texture. For the infusion, 0.7 g of the powdered leaves was mixed with 100 mL of distilled water, then heated to a boiling point (95–100 °C) and stirred for 15 min. Afterward, the infusion was filtered through cotton gauze to remove any solid particles. The filtrate was concentrated using a vacuum rotary evaporator (bath adjustment: 40 °C, rotation: 50 rpm, pressure: ∼15 psi, and condenser: 4 °C) to remove the water and subsequently freeze-dried to eliminate any water traces. The yield of the MO infusion powder was determined as a percentage (*w*/*w*) and stored in a silicone desiccator until further use. The lyophilization process was established for two key reasons: first, to obtain more consistent chemical profiles of the infusion, and second, to allow for better control over the amount of lyophilized powder for precise administration in the animal model [34–36]. This approach ensured more accurate dosing consistency and reliability in the infusion preparation [33]. Preliminary phytochemical characterization and chromatographic profiles, including thin-layer chromatography (TLC), infrared (IR) spectroscopy, and ^1^H nuclear magnetic resonance (NMR), were conducted under similar conditions to those reported previously [33].

The authors state that the scientific conclusions are unaffected. This correction was approved by the Academic Editor. The original publication has also been updated.

## Text Correction 2

During the editorial process, several lines of text were inadvertently omitted. A paragraph has now been added to clarify the relationship between the current study [1] and a previously published work [2]. Both investigations were carried out simultaneously under the same housing conditions, ethical approvals, and control group protocols, which explains why certain values corresponding to the control groups (Balanced diet and Negative control) coincide between the two studies.

However, the experimental designs and treatment objectives were distinct: the 2024 publication evaluated the therapeutic effects of *Moringa oleifera* (MO) [2] infusion or extract in disease-established models, whereas the present 2025 study investigated its prophylactic role in a preventive model. This clarification has been incorporated into Section 2.7 to ensure methodological transparency and to prevent any potential misinterpretation regarding the independence of the experimental data. These textual adjustments do not affect the study’s data, results, or conclusions.

A correction has been made to Section 2.7. Prophylactic Animal Model Design for MO Infusion, Paragraph 1:

*2.7. Prophylactic Animal Model Design for MO Infusion*

In this study, conditions similar to those used in a previous investigation examining the effects of Moringa oleifera (MO) were followed [33]. The current preclinical trial was conducted concurrently with another study involving groups of individuals with established disease (NAFLD) to evaluate the short-term effects of MO, the findings of which have been previously reported [33]. Due to the similarity between the present investigation and the referenced study, some values corresponding to the “control or reference groups” (healthy subjects or untreated NAFLD models) may overlap between both studies.

However, the current study incorporated the following specific adjustments: Male BALB/c mice (Envigo, Indianapolis, IN, USA), aged between 4 and 6 weeks, with an initial weight ranging from 22 to 25 g, were utilized. Each group consisted of 11 mice. The mice were housed under sterile conditions in filter-topped cages to minimize exposure to pathogens, with a maximum of 5 mice per cage. The environment was controlled with a 12 h:12 h light/dark cycle, a temperature of 23 °C, and 50% humidity. They had ad libitum access to food and water. National and international guidelines for laboratory animal care were strictly followed. Prior to the commencement of the experiment, the mice underwent a 7-day acclimatization period. All procedures adhered to national and international ethical standards for preclinical research. The study was approved by the Research Ethics Committee of the Colima State Cancer Institute, Colima, Mexico (Protocol Number: CEICC-240818-ETAMORI-010).

Animal handling followed institutional guidelines, the Mexican official norm for laboratory animals (NOM-062-ZOO-1999), and the Guide for the Care and Use of Laboratory Animals published by the National Academy of Sciences (2011) [51]. Euthanasia was performed according to the American Veterinary Medical Association (AVMA) Guidelines for the Euthanasia of Animals: 2020 Edition [52].

The authors state that the scientific conclusions are unaffected. This correction was approved by the Academic Editor. The original publication has also been updated.

## Figures and Tables

**Figure 6:**
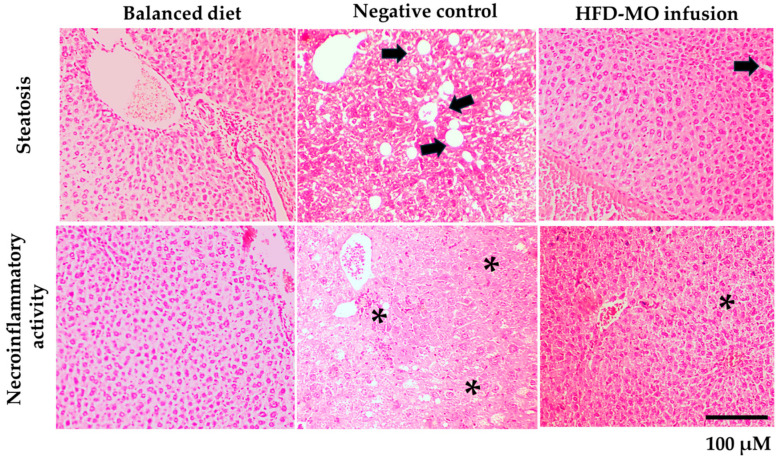
Representative images of the liver show lower steatosis and inflammation among the HFD group treated with MO infusion, compared with the HFD- placebo group. Liver tissues in the balanced diet group show no pathological changes. As an illustration, the black arrows (→) show an area of steatosis, while the asterisks (*) show the presence of inflammatory cells. Images magnified by ×200.
