# Supplementary material for: Moringa oleifera Leaf Infusion as a Functional Beverage: Polyphenol Content, Antioxidant Capacity, and Its Potential Role in the Prevention of Metabolopathies
Source: Life (Basel). 2025 Apr 11;15(4):636. doi: 10.3390/life15040636 (PMC12028896; doi:10.3390/life15040636)
Supplement: Supplementary file 1 [file life-15-00636-s001.zip › Table S1.pdf]

Table S1. Intake and body weight of the experimental groups

	Month	Balanced diet	Negative control	HFD-MO infusion	p ANOVA
Body weight (g)	0	24.57±1.9	24.34±1.8	24.10±2.0	0.976
	1	28.36±2.4	30.21±2.2	26.14 ±1.4[c]	0.025
	2	28.51±2.8	30.59±2.2	26.22±1.4 [c]	<0.001
	3	28.61±2.8	30.93±2.2	26.33±1.4 [c]	<0.001
	4	28.83±2.6	31.37±2.1[a]	26.22±1.5 [c]	<0.001
	5	29.18±2.3	31.83±2.2[a]	25.18±1.6 [c]	<0.001
	6	29.34±2.2	32.00±2.2[a]	21.39±0.9[bc]	<0.001
	7	29.51±2.3	33.08±2.1[a]	21.04±0.9[bc]	<0.001
	8	29.95±2.2	36.12±2.1[a]	20.60±1.0[bc]	<0.001
Food intake (g)	0	2.90±1.0	3.00±0.9	2.88±1.2	0.998
	1	3.43±1.4	5.56±1.6	4.88 ±1.1	0.625
	2	4.02±1.0	6.30±1.2	5.10 ±2.1	0.320
	3	4.67±1.8	7.09±1.0	5.30±1.0	0.058
	4	5.32±0.6	7.46±2.4[a]	5.44±1.2	0.030
	5	5.87±1.3	7.69±1.9[a]	5.56±1.0 [c]	<0.001
	6	6.17±1.2	8.20±2.0[a]	5.44±1.3[bc]	<0.001
	7	6.49±2.0	9.5±1.1[a]	4.91±1.0 [bc]	<0.001
	8	7.54±1.2	11±2.9[a]	4.75±2.0[bc]	<0.001
Food intake (kcal)	0	8.99±3.1	13.50±4.0	12.96±5.4	0.756
	1	10.63±4.3	25.02±7.2[a]	21.96 ±4.9	<0.001
	2	12.46±3.1	28.35±5.4[a]	22.95 ±9.4	<0.001
	3	14.47±5.5	31.90±4.5[a]	23.85±4.5	<0.001
	4	16.49±1.8	33.57±9.8[a]	24.48±5.4	<0.001
	5	18.19±4.0	34.60±8.5[a]	25.02±4.5 [c]	<0.001
	6	19.12±3.7	36.90±9.0[a]	24.48±5.8 [c]	<0.001
	7	20.11±3.2	42.75±4.9[a]	22.09±4.5 [c]	<0.001
	8	23.37±3.7	49.50±8.5[a]	21.37±3.5[c]	<0.001

All values are expressed as Mean ± SEM, (*n*=11), differences from Tukey`s post hoc analysis are marked when they are significant: [a]p= <0.05 when compared balanced diet vs HFD-placebo, [b]balanced diet vs HFD-MO infusion, [c]Negative control vs HFD-MO infusion (n= 11 per group).
