# Supplementary figures and images for: Moringa oleifera Leaf Infusion as a Functional Beverage: Polyphenol Content, Antioxidant Capacity, and Its Potential Role in the Prevention of Metabolopathies
Source: Life (Basel). 2025 Apr 11;15(4):636. doi: 10.3390/life15040636 (PMC12028896; doi:10.3390/life15040636)

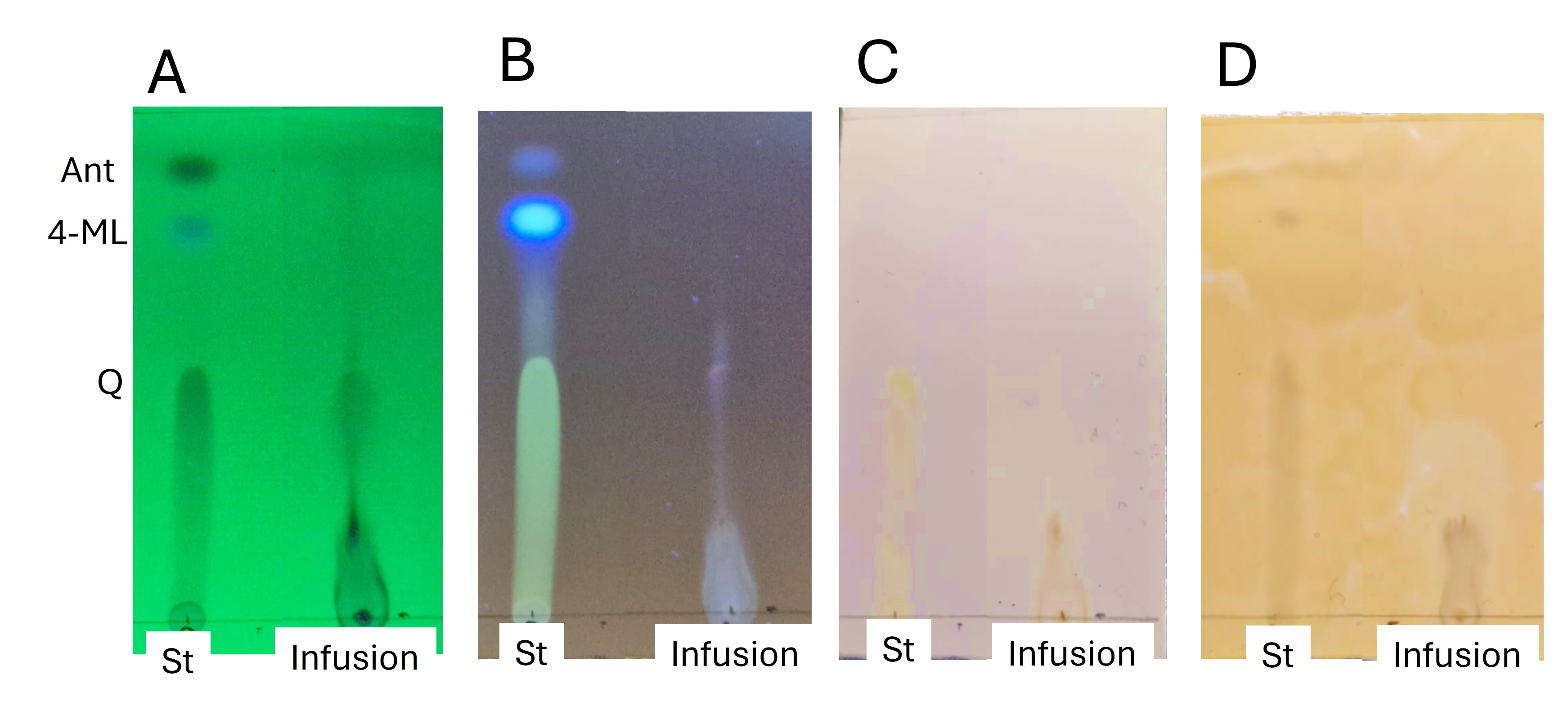

Supplement: Supplementary file 1 [file life-15-00636-s001.zip › Figure S1.jpg]

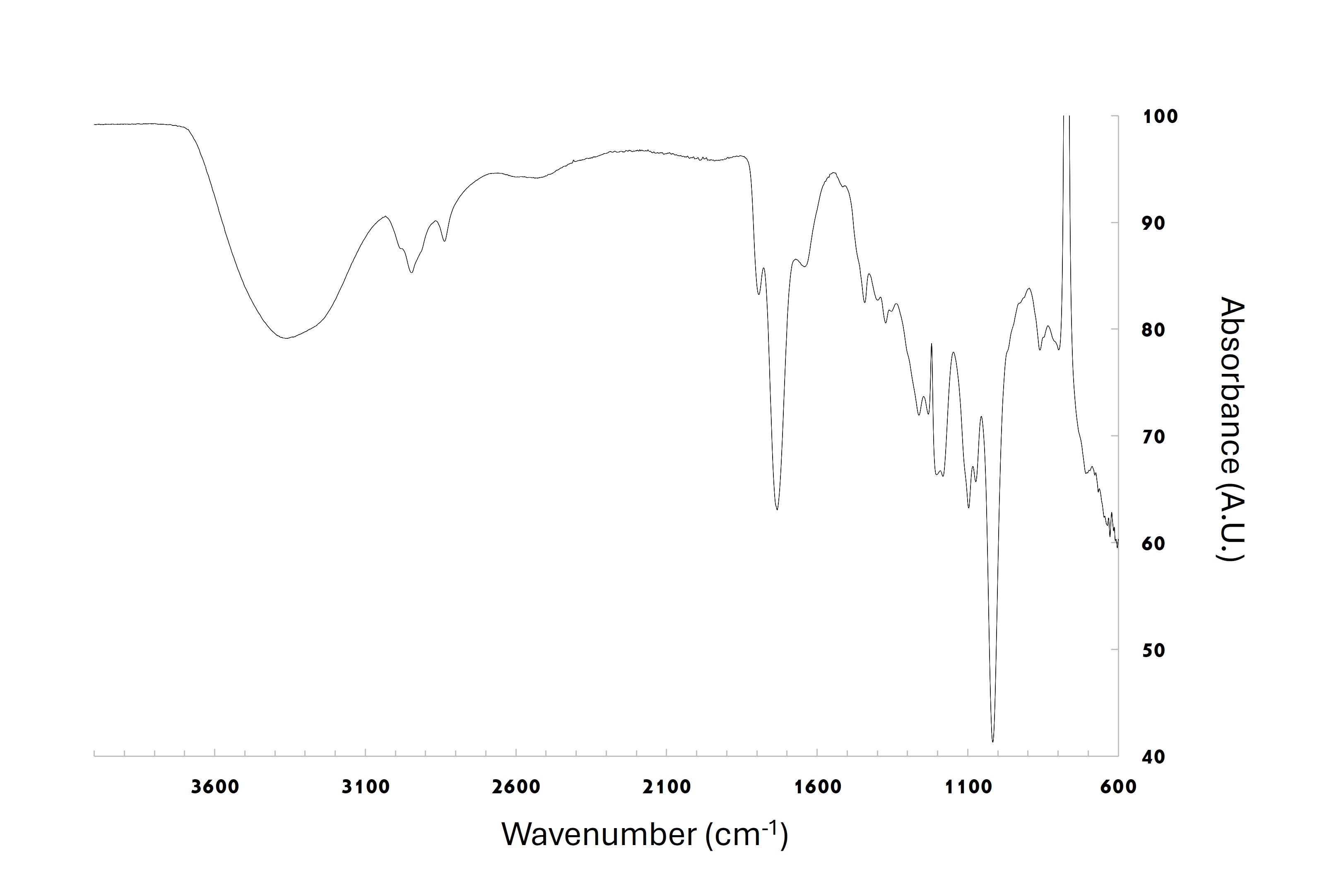

Supplement: Supplementary file 1 [file life-15-00636-s001.zip › Figure S2.jpg]

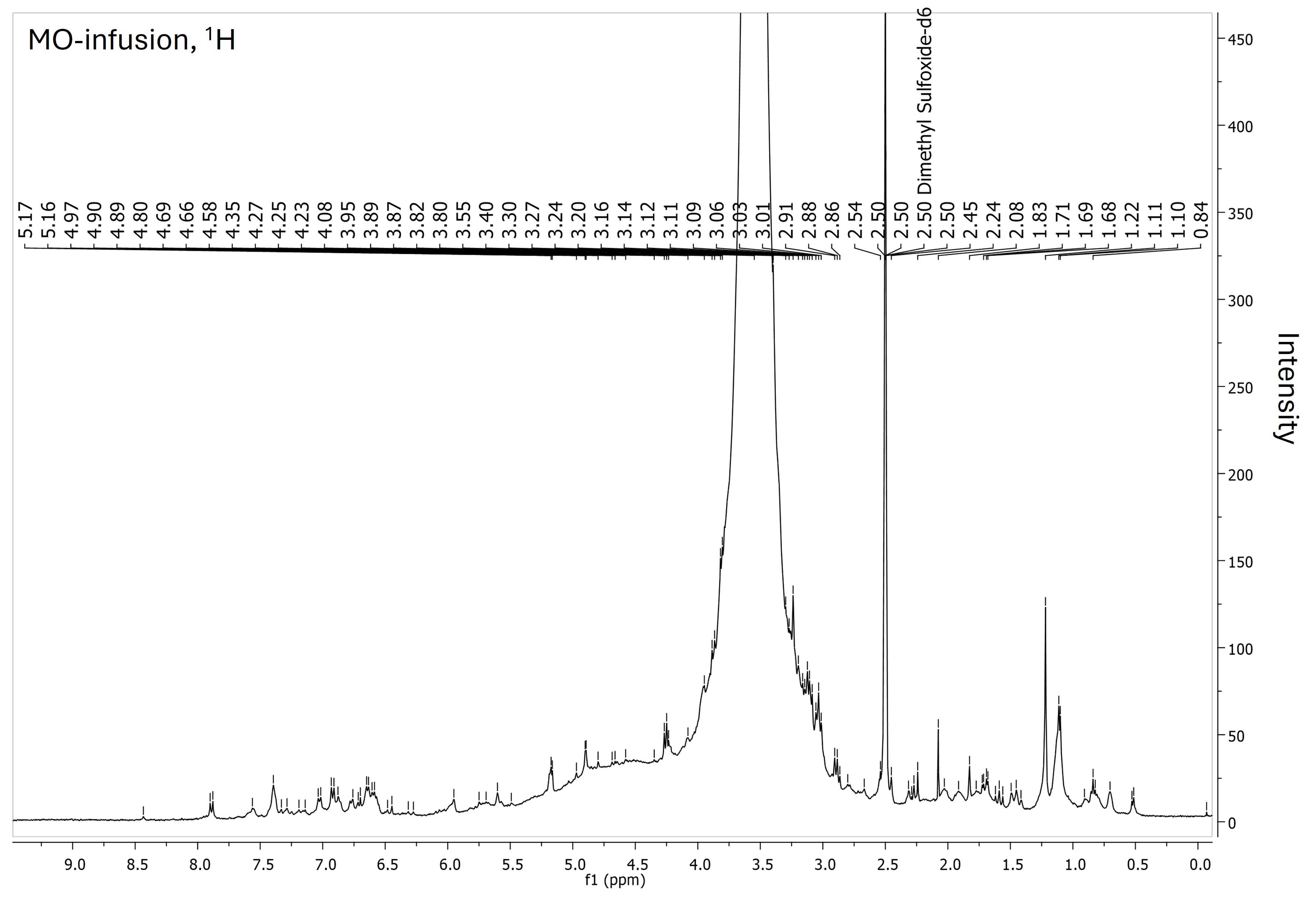

Supplement: Supplementary file 1 [file life-15-00636-s001.zip › Figure S3.jpg]
